# The Role of CSR Engagement in Customer-Company Identification and Behavioral Intention During the COVID-19 Pandemic

**DOI:** 10.3389/fpsyg.2021.721410

**Published:** 2021-08-12

**Authors:** Yanqin Wu, Wenzhong Zhu

**Affiliations:** ^1^School of English for International Business, Guangdong University of Foreign Studies, Guangzhou, China; ^2^School of Business, Guangdong University of Foreign Studies, Guangzhou, China

**Keywords:** CSR engagement, behavioral intention, C-C identification, Chinese shared brands, COVID-19 pandemic

## Abstract

The global coronavirus pandemic has reignited a strategic debate among the business community of the necessity for corporate social responsibility (CSR) engagement in the ever-dynamic social media. Considering the global economic downturn introduced by the COVID-19 pandemic, the present research is devoted to investigating whether CSR engagement in social media can help DiDi (a Chinese shared brand) at stake survive this overwhelming crisis. A theoretical model proposed to describe the hypothesized relationships was tested by a Structural Equation Modeling technique through the empirical online questionnaire. Through findings, we demonstrated that there was a positive relationship between CSR engagement of DiDi on WeChat, customer–company identification (C–C identification), and behavioral intention [purchase intention, brand loyalty, and e-word-of-mouth (eWOM)] of customers. With attention to psychological influence, our empirical statistics also evidenced the mediating role of C-C identification between CSR engagement and behavioral intention of customers. This study highlights the significant role of CSR engagement in a critical period theoretically and offers businesses more open innovation strategies to compete against the COVID-19 pandemic-induced market downturn.

## Introduction

The practice of corporate social responsibility (CSR) with the actual positive impacts has been increasingly incorporated into the strategic movement of business operations (Lii and Lee, 2012; Hu et al., 2019). The European Commission (2011) theorizes that CSR initiatives refer to the integration of the companies of social and environmental concerns into their business operations. In addition to CSR initiatives, successful CSR engagement also requires the company to effectively communicate their CSR activities with their stakeholders through various platforms (Fatma et al., 2020). In general, CSR engagement by business organizations is supposed to effectively affect the behavioral intention of the consumers, which can eventually contribute to the increased economic benefits (Sharma et al., 2018; Puriwat and Tripopsakul, 2021). In regardless of the favorable outcomes of CSR engagement, discrepancies occur in the academic world that whether CSR engagement is of necessity in a critical period, e.g., COVID-19 (Han, H. et al., 2020; Bae et al., 2021; Puriwat and Tripopsakul, 2021). Since this type of activity requires companies' investment of their limited available capitals that are might needed by other promotional activities as well.

Social media are experiencing a booming development with the advent of information and technology, drastically changing the communication landscape where people interact with each other. Owing to its easy accessibility, people are increasingly involving themselves in this environment, contributing to the great prevalence of social media (Du and Vieira, 2012; She and Michelon, 2019). In particular, the sudden outbreak of the COVID-19 pandemic has furthered a digitalized transformation of human interaction where there is a reported increase in the users of social media (Lep et al., 2020; Vanko et al., 2021). Meanwhile, social media can be utilized by companies to extend businesses to a wider audience with a global reach and affect the behavioral intention of consumers while engaging in their CSR initiatives (Bialkova and Te Paske, 2020; Kuhzady et al., 2020). However, through a review of a study, the academy concerning CSR practices lags behind the practices of CSR engagement through social media (Kesavan et al., 2013; Chu et al., 2020). Among these studies, there is also a lack of explorative insights specifying the underlying psychological mechanism of how CSR engagement affects the behavioral intention of customers online in a specific period (Ahearne et al., 2005; Han, H. et al., 2020). Nonetheless, it has been acknowledged that psychological experience is indispensible for the illustration of human reasoned behaviors (Warshaw and Davis, 1985; Tomczyk et al., 2020).

Shared brands as a new pattern of business operation are reported as seriously short of CSR engagement even when staying in an optimistic context (Bhappu and Schultze, 2019). Toward the conceptualization of this emerging economy, a consensus is that it advocates for peer-to-peer collaborative consumption and better use of idle resource based on advent technology (Hu et al., 2019; Kuhzady et al., 2020). Regardless of being viewed as a pathway to sustainability, criticism on shared brands claims that its unregulated system backfires on a sustainable ideal, contributing to even serious over-consumption (Martin, 2016; Bhappu and Schultze, 2019). Nonetheless, these new consumption platforms based on collaborative consumption are heralded to become a dominant business system in the future (Heinrichs, 2013; Martin, 2016). Regarding the contradictory nature of shared brands, to the best of our knowledge, there are no studies that explored the CSR engagement by shared brands to find out the possible solutions (Bhappu and Schultze, 2019; Hu et al., 2019).

In a nutshell, to fill these identified voids, this study dissected the psychological process of customers toward the effects of CSR engagement by shared brands at a time that the communication landscape has been transforming into an ever-digitalized one during and after the COVID-19 pandemic. More precisely, in light of the changes happening in the way people interact with others during the coronavirus pandemic, this study is devoted to investigating whether and how CSR engagement of DiDi (a Chinese shared brand) on WeChat can exert its impacts on the behavioral intention of customers. Besides, given the dominant role of customer–company identification (C-C identification) in exploring the psychological state of customers, this study is also concerned with its operation underlying such impact of CSR engagement. Overall, this study in nature focused on the relationships among CSR engagement of DiDi, C–C identification, and behavioral intention of customers in an era of the COVID-19.

## Literature Review

### CSR Engagement of Companies *via* Social Media

Apart from economic agendas, companies nowadays have increasingly assumed much importance on CSR engagement, which is viewed as a strategic movement for their business development (Kesavan et al., 2013; Puriwat and Tripopsakul, 2021). According to Fatma et al. (2020), CSR engagement is related to how companies perform and communicate their CSR-related activities. In this way, on one hand, companies directed at CSR engagement should practice their own CSR initiatives beyond economic benefits (He and Li, 2011; Puriwat and Tripopsakul, 2021). On the other hand, alike CSR initiatives, equally important for companies is to ensure that CSR-related information is communicated to stakeholders, which is referred to as CSR communication (Oh and Ki, 2019; Fatma et al., 2020).

Scholars have evidenced CSR engagement in the study whereby companies can reap a myriad of benefits, for example, enhanced impression management and positive behavioral intention of customers, such as positive word-of-mouth (WOM) (Kesavan et al., 2013; Abitbol and Lee, 2017; Sharma et al., 2018; Puriwat and Tripopsakul, 2021). Most notably, it arises as a heated issue whether these positive effects from CSR engagement can be stimulated as well in a critical period, for instance, the global economic recession introduced by the COVID-19 (Puriwat and Tripopsakul, 2021; Vanko et al., 2021). Therefore, discrepancies occur concerning the necessity of organizational efforts in CSR engagement when the available capitals for economic agendas are simply not enough in times of crisis (Han, H. et al., 2020; Bae et al., 2021).

In the era of web 2.0, there is a paradigm shift that social life has gradually transformed into a digitalized model with the involvement of social media (Vanko et al., 2021). These emerging social platforms exhibit their great potentials to generate more dialogic dynamics through which individuals can have more access to organizational information and even share their attitudes with fewer constraints (She and Michelon, 2019; Fatma et al., 2020). The physical disconnect with the society caused by the coronavirus has further accelerated the use of social media by which people keep in touch with the outside (Lep et al., 2020; Hayes and Clerk, 2021; Puriwat and Tripopsakul, 2021). With regard to the ever-digitalized social life, the emerging social media afford companies more possibilities to promote their CSR-dedicated activities, which, in turn, can help companies maximize their benefits from CSR engagement if used effectively (She and Michelon, 2019; Bialkova and Te Paske, 2020).

As indicated by Chu and Chen (2019), considerable research studies on CSR engagement have majorly discussed it in the traditional environment, for example, corporate websites. Among these studies, the positive effect of such an issue on the behavioral intention of customers has been continuously stressed. Nonetheless, how do social media empirically interplay with CSR engagement has relatively been an under-researched issue in the study regardless of its actual prevalence (Chu and Chen, 2019; Chu et al., 2020; Fatma et al., 2020). Furthermore, there is an identified gap regarding the underlying psychological process of how CSR engagement of companies affects the behavioral intention of consumers in social media (Lii and Lee, 2012; Martínez and Rodríguez del Bosque, 2013; Puriwat and Tripopsakul, 2021). Thus, in light of the increasingly digitalized world, this study investigates the psychological mechanism of whether and how the behavioral intention of consumers can be empirically influenced by the CSR engagement of companies in social media during the damaging COVID-19 pandemic.

### CSR Engagement of Different Brands

Regardless of the general criteria offered to guide CSR engagement of consumers, existing research studies also suggest that discrepancies may occur while being examined in different industrial contexts (Fatma et al., 2018; White and Alkandari, 2019; Chu et al., 2020). More specifically, scholars perceive CSR engagement in nature as a context-based notion that cannot be separated from contextual considerations across culture and industry. In CSR academia, there is a mixed finding regarding the influence of CSR engagement on the behavioral intention of consumers where companies from different countries and industries had been examined (Fatma et al., 2018; Chu et al., 2020).

The digitalized life supported by advanced information and technology has nudged the development of shared brands, that is, not merely limited to information sharing (Kuhzady et al., 2020). The conceptualization of shared brands indicates further openness through which individuals can enjoy and participate in the production of service and products, such as houses, cars, and charge pals (Martin, 2016). The new pattern of shared brands motivated by sustainability is experiencing remarkable growth in the global market, even threatening the existence of traditional industries and heralding a new way to sustainability (Heinrichs, 2013; Martin, 2016). Nonetheless, amid great commercial achievement, the pattern of shared brands has also been continuously critiqued as disruptive and sometimes destructive toward society, which highlights solutions from academic discussions (Bhappu and Schultze, 2019; Galati et al., 2019). One of the typical case is the safety problems of Airbnb introduced by its unregulated system, a peer-to-peer platform through which individual can enjoy and provide apartment rental services (Martin, 2016). Toward the current dilemma confronted by shared brands, one of the solution advanced by researchers is CSR engagement whereby practitioners can optimize business activities of the shared brands (Bhappu and Schultze, 2019; Hu et al., 2019).

In terms of the context-based characteristics of CSR engagement, extant studies have typically considered its effect on the behavioral intention of consumers in the context of audit institution (She and Michelon, 2019), bank (Brammer et al., 2007; Puriwat and Tripopsakul, 2021), and hospitality (Fatma et al., 2018; Han S. H. et al., 2020; Puriwat and Tripopsakul, 2021). Despite the acknowledged importance of CSR engagement, there are few existing studies that have focused on the role of CSR engagement of shared brands (Hu et al., 2019). Given the increasing prevalence of shared brands, this study is devoted to discussing the CSR engagement of Chinese shared brands in such a critical period as the pandemic COVID-19.

## Hypothesis Development and Conceptual Framework

### CSR Engagement and C–C Identification in Social Media

Customer–company identification dependent on social identity theory generally illustrates the psychological state of consumers toward a company (Atakan-Duman and Ozdora-Aksak, 2014; Fatma et al., 2018). Based on social identity theory, it claims that people tend to locate and define themselves into specific social categories after a cognitive categorization process, when they find some similarities and overlap with the compared group (Tajfel and Turner, 1979; Bi et al., 2018). In this way, C–C identification can be ascribed as a specific social categorization after which customers attempt to build a strong emotional attachment toward a company (Fatma et al., 2018, 2020; Chu and Chen, 2019). When customers identify with their target groups, a sense of belonging will be instilled into their mind (Chu and Chen, 2019).

Among the special projects to identify probable consumers, CSR engagement is supposed to be of great value during such a process (Bhattacharya and Sen, 2003; Lii and Lee, 2012; Fatma et al., 2020). Through CSR engagement, companies, in general, are showing their commitment to the welfare of society, which, in turn, is more likely to satisfy the self-need of customers. In a similar vein, the previous studies have demonstrated that C–C identification can be stimulated when an organization is perceived as a socially responsible citizen by their customers (Bhattacharya and Sen, 2003; Lii and Lee, 2012). In comparison to the traditional communication landscape, the digitalization of social life empowers people to be more actively involved in the activities of companies instead of mere passive recipients (Chu and Chen, 2019; Fatma et al., 2020). Therefore, from a theoretical perspective, companies through these emerging social media (e.g., *Twitter, Weibo*, and WeChat *Official Account*) can increase consumer awareness about their CSR engagement effectively, which, in turn, can have a positive influence on C–C identification in such context.

For the above discussion, it was expected in this study that CSR engagement of shared brands in social media could affect their C–C identification during the pandemic COVID-19, which is represented as H1:

**Hypothesis 1**: There is a positive relationship between CSR engagement of the shared brands in social media and the C–C identification during the pandemic COVID-19.

### Customer–Company Identification and Behavioral Intention of Consumers in Social Media

Behavioral intention as a dominant motivator of human reasoned behavior is defined as “*the degree to which a person has formulated conscious plans to perform or not perform some specified future behavior* (Warshaw and Davis, 1985, p.124). Similarly, Fishbein and Ajzen (1975) claimed that behavioral intention as a psychological outcome can be a strong predictor of the actual future behavior of an individual. Toward the regular items adopted for the measurement of the behavioral intention of customers in social media, existing studies suggest that three constructs should be incorporated: brand loyalty, purchase intention, and e-word-of-mouth (eWOM) (Chang, 2016; Chu and Chen, 2019). Brand loyalty refers to the deeply held commitment of customers toward a company, its service, or product, which can increase the economic profit of companies, especially in a critical period (Fatma et al., 2018; Han et al., 2019). In view of the overwhelming coronavirus-induced economic downturn, brand loyalty of customers then should be highlighted as a crucial asset that assists companies in competing against the competitive but shrinking market in the unfavorable external environment. It is noteworthy that Fatma et al. (2018) suggested that the evaluation of brand loyalty necessitates a two-fold consideration including attitudinal and behavioral loyalty. The loyalty of the consumers at the level of attitude on social media refers to a favorable attitude toward the company, which can lead to positive eWOM (Bhattacharya and Sen, 2003). Meanwhile, behavioral loyalty generally emphasizes the practical effects on purchase intention, which results in the monetary spending behaviors of patronages (Sharma et al., 2018; Chu and Chen, 2019).

In view of reasons behind these behavioral intentions, Ahearne et al. (2005) through empirical studies have evidenced that C–C identification is in essence an underlying psychological driver. With stronger C–C identification, it is suggested that customers will emotionally attach themselves to the company, which, in turn, can promote them to develop and safeguard this company in social media (Bhattacharya and Sen, 2003; Chu et al., 2020). In this regard, customers identified with a company will show more willingness to build long-term and loyal relationship with it (Bhattacharya and Sen, 2003; Fatma et al., 2020), to make repurchases (Ahearne et al., 2005; Martínez and Rodríguez del Bosque, 2013), and to disseminate its positive WOM voluntarily in social media (Chu and Chen, 2019).

For this, our study argued that with stronger C–C identification, Chinese shared brands could impact the behavioral intention of their customers positively during the COVID-19 pandemic, which was proposed as H2. It further postulated that brand loyalty plays a dominant role in the framework of behavioral intention of customers, which constitutes hypothesis 3. More precisely, it was indicated that brand loyalty of Chinese shared brands is positively related to purchase intention and eWOM of their customers in social media.

**Hypothesis 2:** There is a positive relationship in social media between C–C identification and (a) purchase intention, (b) brand loyalty, and (c) eWOM in social media of the shared brands during the pandemic COVID-19.

**Hypothesis 3:** There is a positive relationship in social media between brand loyalty of the shared brands and (a) purchase intention and (b) eWOM in social media during the pandemic COVID-19.

### The Mediating Effect of C–C Identification Between CSR Engagement and Behavioral Intention in Social Media

Based on social identity theory, CSR engagement has shown its great potential to get C–C identification with which customers have the propensity to build an emotional and psychological link with the company (Fatma et al., 2018, 2020; Chu and Chen, 2019). Subsequently, customers with a strong C–C identification are meanwhile prone to extra-role behaviors that are “directed toward preserving, supporting, and improving the organization” (Ahearne et al., 2005, p.577), for instance, brand loyalty and eWOM (Bhattacharya and Sen, 2003).

Prior studies have evidenced that the behavioral intention of customers is a significant analytic point to illustrate the outcome of CSR engagement of business organizations (Zeithaml et al., 1996; Fatma et al., 2018; Puriwat and Tripopsakul, 2021). Moreover, C–C identification as an antecedent can offer a more detailed interpretation of the psychological process of how the behavioral intention of customers is gradually formulated (Bhattacharya and Sen, 2003; Martínez and Rodríguez del Bosque, 2013). Therefore, this study aims to externalize the internal logic between the CSR engagement and the behavioral intention of customers, where C–C identification is supposed to play a mediating role from the perspective of psychological analysis. In this regard, the following hypothesis was proposed:

**Hypothesis 4:** C–C identification mediates the relationship between CSR engagement of the shared brands in social media and behavioral intention during the pandemic COVID-19.

**Figure 1:**
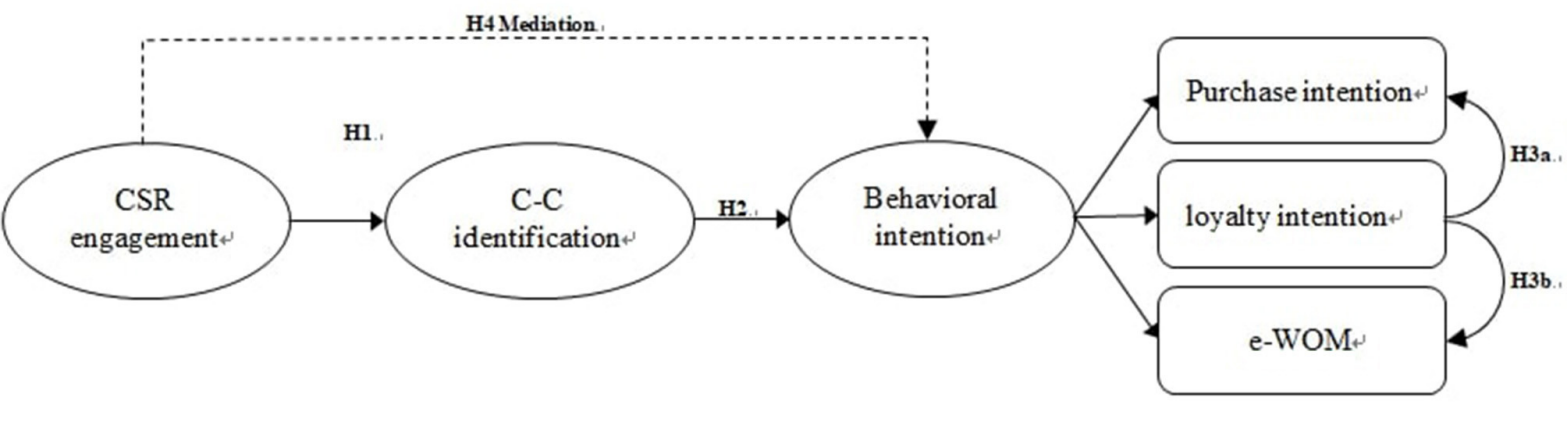
The proposed theoretical framework and hypothesis.

Based on the above arguments, this study then proposed a research framework that describes the hypothesized relationship among CSR engagement of shared brands, C–C identification, and behavioral intention in social media. Figure 1 presents the theoretical framework in this study.

## Methodology

The advent of information and technology is giving rise to a new pattern of business operation as shared brands and human interaction on social sites, such as Facebook and WeChat (Kuhzady et al., 2020). Social media have experienced a booming development in China where the Chinese companies and customers are becoming more active in the virtual community (Chu and Chen, 2019). Furthermore, the vitality of social media has ever been strengthened while people are encouraged in home quarantine during the COVID-19. Of the various social media in China, WeChat is one of the most popular platforms in the circumstance that Facebook and Twitter are not accessible to the Chinese. According to the financial report of Tecent, there was a sum of 12.25 billion users on WeChat up to the year 2020 (Tecent Holdings Limited, 2021). Compared to the statistics of 2019, it indicates an ever-digitalized evolution of the communication landscape during the coronavirus crisis. Together with the great economic performance Chinese shared brands have achieved, this study, therefore, is committed to the effect of its CSR engagement through WeChat on the behavioral intention of customers during the COVID-19. Of the various sharing industries in China, DiDi as a Chinese car-sharing platform was finalized for the research design that featured on the top 10 list of global unicorns maintained by *CB Insights* (https://www.cbinsights.com/research/infographics/) in 2020.

An online survey organized through Sojump (http://www.sojump.com/) was distributed to gather more responses from the target groups, lasting from January 12, 2021 to April 31, 2021. To reduce common method bias, an anonymous promise was also guaranteed during the whole study. After data filtering, 241 valid surveys were received in this study, which is considered as adequate for the analytic procedure (Kline, 2011).

### Research Design and Measurement Generation

To obtain an initial insight of subordinate measurement items under each construct, the researcher reviewed the study related to CSR engagement, C–C identification, and behavioral intention. A draft questionnaire was developed incorporating the most prevalent measurement items in extant studies, which can strengthen the content validity of the research design (Shah and Ward, 2007; Kuhzady et al., 2020). The CSR engagement in the questionnaire was assessed by a three-item scale offered by Martínez and Rodríguez del Bosque (2013). C–C identification was evaluated by a four-item scale from Mael and Ashforth (1992). Brand loyalty was measured by a four-item scale from the study of Zeithaml et al. (1996). Purchase intension, meanwhile, was calculated with a three-item scale from Kang and Hustvedt (2014). eWOM was then measured by a three-item scale, adopted from Chu and Chen (2019). In light of the impact of industrial difference on the measurement items, a deep interview with two relevant experts was conducted whose suggestions were incorporated to refine the measurement contents. In this way, 17 items were determined as the initial measurement tools of our own research model.

After that, this study adopted a 7-point Likert scale to evaluate all items for a comprehensive understanding ranging from strongly disagree (1) to strongly agree (7). Before the main survey was administrated, this study also performed a pilot study with a sample size of 20 participants to confirm the suitability and understandability of the questionnaire. Through analysis, its result showed that there were two items from purchase intention and brand loyalty with the factor loading < 0.5, which were then removed from the final questionnaire. Table 1 summarizes the final structure of our measurement construct, which in total incorporated 15 measurement items.

**Table 1:** Description of measurement constructs and items.

**Constructs**	**Items**	**Observed variables**	**Source**
CSR engagement	CSR1	During the pandemic COVID-19, DiDi is committed to donation activities.	Martínez and Rodríguez del Bosque, 2013
	CSR2	During the pandemic COVID-19, DiDi uses part of its budget to assist the community in which it operates.	
	CSR3	During the pandemic COVID-19, DiDi is an environmentally responsible corporate.	
Customer-company identification	CCI1	When someone praises DiDi, it feels like a personal compliment.	Mael and Ashforth, 1992
	CCI2	When someone criticizes DiDi, it feels like a personal insult.	
	CCI3	I'm very interested in what others think about DiDi.	
	CCI4	When I talk about DiDi, I usually say “we” rather than “they.”	
Brand loyalty	BL1	I prefer to use DiDi as my first choice as compared to other car-hailing apps.	Zeithaml et al., 1996
	BL2	I will consider DiDi as my main car-hailing app in the next few years.	
	BL3	It would be costly of time and money to end my relationship with DiDi.	
Purchase intention	PI1	I will consider using DiDi the next time I need car-hailing service.	Kang and Hustvedt, 2014
	PI2	I will use DiDi the next time I need car-hailing service.	
E word-of-mouth	eWOM1	I am willing to sat positive things of DiDi in social media.	Chu and Chen, 2019
	eWOM2	I will recommend DiDi to my friends and relatives in social media.	
	eWOM3	If my friends are looking for car-hailing service, I will encourage them to be a customers of DiDi.	

The main content of the questionnaire was organized into two parts. The first part was majorly concerned with a general demographic picture of the respondents, including their age, gender, occupation, and city. The second part of the survey was designed to explore the attitudes of the participants toward each measurement item based on the 7-point Likert scale, which were tested as follows.

### Sample and Data Collection

To sample which respondents to be included in this study, a purposive sampling technique was adopted to assure their representativeness. Accordingly, to more precisely describe how the CSR engagement of DiDi is operationalized on WeChat, the questionnaire also set several screening questions that remove respondents from being not involved in this process. To guarantee the representativeness of the respondents, interviewees at first were required to answer three questions, (1) Have you ever used DiDi in your life through WeChat small program? (2) Are you a follower of WeChat official account of DiDi? (3) Are you a follower of WeChat channel account of DiDi? Only respondents who answered “yes” toward either question were allowed moving on to the main questionnaire. An operational definition of CSR and its empirical cases was also provided at the beginning of the questionnaire. More precisely, participants read: “CSR is an organizational effort into social and environmental issues that goes beyond their economic profit. For example, Meituan offers contactless delivery service during the COVID-19, ensuring the safety of their customers and man for delivery. Another one is Alipay issues the Ant Forest project designed for reducing carbon emission.” After that, the participants were required to recall their experience concerning with how they perceive the CSR-related information of DiDi on WeChat while filling the questionnaire.

## Results

### Sample Profile

Table 2 summarizes the descriptive demographic information of the respondents in the main survey through SPSS 22. Among them, it could be observed that 40.66% of them were men, and 59.34% of them were women. In all, the majority of them were students (41.91%) and working employees (30.29%).

**Table 2:** Profile of the respondents.

**Item**	**Description**	**Sample**	**%**
Gender	Male	103	40.66
	Female	146	59.34
Age (years)	18–24	124	51.45
	25–34	57	23.65
	35–44	34	14.11
	45–54	18	7.47
	55 or above	8	3.32
Occupation	Individual business	23	9.54
	Freelancer	10	4.14
	Working employees	73	30.29
	Students	101	41.91
	Others	34	14.11
City	Guangdong	80	33.2
	Hubei	69	28.63
	Hunan	43	17.84
	Jiangsu	32	13.28
	Others	17	7.05

### Measurement Model

Before testing the proposed theoretical model, all the constructs and their corresponding subscales were assessed to ensure their validity and reliability with the assistance of SPSS 22 and AMOS 26.

#### Reliability Test

To test the scale reliability of each construct, the value of Cronbach's alpha was calculated through SPSS 22, whose results are presented in Table 3. All coefficient alphas ranging from 0.823 to 0.94 were far higher than the recommended value of 0.7 according to Nunnally (1978), indicating the adequate consistency of the internal items in this study and the desired reliability of the measurement model.

**Table 3:** Reliability test.

**No**.	**Constructs**	**Items**	**Cronbach's alpha**
1	CSR engagement	3	0.929
2	C-C identification (CCI)	4	0.94
3	Brand loyalty (BL)	3	0.883
4	Purchase intention (PI)	2	0.823
5	E-word of mouth (eWOM)	3	0.916

#### Validity Test

Apart from reliability test, the researcher also examined the scale validity from two aspects: content validity and construct validity. With regard to the content validity, the measurement development was first based on a review of a mature study from which the most prevalent scales were adopted (Shah and Ward, 2007; Kuhzady et al., 2020). Furthermore, in consideration of the industrial and national difference, several amendments were conducted through the interview and pilot study.

An exploratory factor analysis (EFA) together with a confirmatory factor analysis (CFA) was performed to examine the construct validity in this study. In the phrase of EFA, the KMO sampling adequacy and Bartlett's test of Sphericity were first assessed. The value of KMO = 0.939 was well above the threshold value of 0.70 according to Sreejesh and Mohapatra (2013). Meanwhile, the results from Bartlett's test also indicated good significance based on the value of approximate chi-square = 3615.02 and *p* = *0*. All statistic data hence showed favorable suitability of the present measurement model for path analysis.

To further confirm the results of EFA, a CFA examining the convergent validity and discriminant validity was also performed, where most indexes indicated a satisfactory fit of the measurement model (CMIN = 133.523, df = 77, CMIN/df = 1.734, GFI = 0.932, RMSEA = 0.055, CFI = 0.984, and NFI = 0.964). According to Awang (2015), there are normally three indexes of convergent validity while the correlations inside each construct is assessed: factor loading, average variance extracted (AVE), and composite reliability (CR). For a measurement model with good convergent validity, it is suggested that the cutoff value should be higher than 0.7, 0.5, and 0.7, respectively. With regard to their values presented in Table 4, it implies that the measurement scale reached a satisfactory convergent validity in this study.

**Table 4:** Descriptive statistics of convergent validity.

**Constructs**	**Item**	**Standardized factor loading**	**SE**	**CR**	**AVE**
CSR engagement	CSR1	0.919^***^	-	0.93	0.817
	CSR2	0.931^***^	0.045		
	CSR3	0.862^***^	0.05		
CCI	CCI1	0.928^***^	-	0.944	0.812
	CCI2	0.959^***^	0.036		
	CCI3	0.904^***^	0.043		
	CCI4	0.782^***^	0.046		
BL	BL1	0.906^***^	-	0.89	0.729
	BL2	0.768^***^	0.059		
	BL3	0.89^***^	0.048		
PI	PI1	0.88^***^	-	0.834	0.718
	PI2	0.802^***^	0.052		
eWOM	eWOM1	0.871^***^	-	0.919	0.792
	eWOM2	0.926^***^	0.056		
	eWOM3	0.868^***^	0.056		

*SE, standard error*.

*** *p < 0.01*.

In comparison to the convergent validity, the discriminant validity emphasizes the uniqueness of each construct from its other counterparts. The good discriminant validity, in general, necessitates a higher value of its square root of AVE of one construct compared with its correlations with other constructs. The descriptive statistics in Table 5 illustrates the good discriminant validity among constructs in this study.

**Table 5:** Descriptive statistics of the discriminant validity.

	**CSR engagement**	**CCI**	**BL**	**PI**	**eWOM**
CSR engagement	0.904				
CCI	0.712	0.901			
BL	0.66	0.775	0.854		
PI	0.661	0.765	0.796	0.847	
eWOM	0.629	0.707	0.695	0.632	0.89

### Hypothesis Testing

To complete the assessment of the measurement model, a structural equation modeling (SEM) technique was applied to test the proposed hypotheses in the research model. From various indexes, there was an observed satisfactory fit of the research model to the data (CMIN = 184.231, df = 81, CMIN/df = 2.274, GFI = 0.906, RMSEA = 0.073, CFI = 0.971, and NFI = 0.951). Table 6 summarizes the results of hypothesis testing from SEM.

**Table 6:** Results of hypotheses testing.

**Hypothesized path**	**Standardized path coefficient (β)**	**t-value**	**SE**	**Supported**
H1a CSR engagement—CCI	0.760^**^	14.089	0.068	Yes
H2a CCI—PI	0.383^**^	4.834	0.056	Yes
H2b CCI—BL	0.812^**^	15.358	0.046	Yes
H2c CCI—eWOM	0.397^**^	4.367	0.076	Yes
H3a BL—PI	0.588^**^	7.099	0.068	Yes
H3b BL—eWOM	0.425^**^	4.565	0.090	Yes

** *p < 0.01*.

Overall, the standardized value from path analysis suggested that all the proposed hypotheses in the research model were supported by regression results. In light of Hypothesis 1, from data it is suggested that the CSR engagement of DiDi on WeChat significantly affected C–C identification (β = 0.745, *p* < 0.01). Meanwhile, the standardized result of path analysis also showed a positive correlation between C–C identification and behavioral intention of customers (H2a: β = 0.383, *p* < 0.001, H2b: β = 0.812, *p* < 0.01, H2c: β = 0.392, *p* < 0.01). Finally, the results of SEM also supported that brand loyalty had a positive impact on the purchase intention of customers (H3a: β = 0.588, *p* < 0.01) and eWOM (H3b: β = 0.425, *p* < 0.01).

In addition to the direct effect examined through path analysis, the casual steps approach and Sobel test were adopted to assess the mediating effect of C–C identification between CSR engagement and three behavioral intention (H4a–H4c). Table 7 shows the results of the evaluation of the hypothesized mediating effects with the demographic information being set as controllable variables. In view of the H4a, first a regression analysis was conducted where CSR engagement and brand loyalty were set as the independent variable and dependent variable, respectively, indicating a significant positive effect of CSR engagement on brand loyalty (Model 1, β = 0.710, *p* < 0.01). Second, the regression coefficient from the independent variable (CSR engagement) and the mediator (C–C identification) showed a significant relationship in Model 7 (β = 1.095, *p* < 0.01). Third, there was also an observed significant positive effect when CSR and C–C identification were processed as independent variables simultaneously and brand loyalty as dependent variable (Model 1, β = 0.249, 0.421, *p* < 0.01). Considering that the regression coefficient of CSR engagement and brand loyalty (0.249) from the third step was smaller than the one (0.710) from the first step, it could be concluded that there was a partial mediating effect of C–C identification between CSR engagement and brand loyalty. Therefore, H4a was supported through the above regression analysis. The same approach was also used to assess H4b and H4c, whose regression results both indicated the partial mediating effects of C–C identification between CSR engagement and purchase intention and eWOM, respectively.

**Table 7:** Results of mediating effect between CSR engagement and behavioral intention.

	**BL**		**PI**		**e-WOM**		**CCI**
	**M1**	**M2**	**M3**	**M4**	**M5**	**M6**	**M7**
Independent variable (CSR engagement)	0.710^**^ (13.534)	0.249^**^ (4.243)	0.362^**^ (13.062)	0.132^**^ (4.133)	0.670^**^ (11.989)	0.273^**^ (4.005)	1.095^**^ (14.766)
Mediating variable (CCI)		0.421^**^ (11.337)		0.210^**^ (10.412)		0.362^**^ (8.394)	
**Control variable**							
Gender	−0.119 (−0.316)	−0.236 (−0.778)	0.045 (0.225)	−0.014 (−0.083)	−0.342 (−0.853)	−0.443 (−1.256)	0.278 (0.522)
Age	−0.027 (−0.127)	0.267 (1.567)	−0.051 (−0.462)	0.096 (1.031)	−0.355 (−1.593)	−0.102 (−0.516)	−0.697^*^ (−2.357)
Occupation	0.232 (1.807)	0.170 (1.639)	0.072 (1.054)	0.040 (0.717)	−0.199 (−1.452)	−0.252^*^ (−2.096)	0.148 (0.816)
City	−0.040 (−0.289)	−0.072 (−0.640)	0.049 (0.659)	0.033 (0.536)	−0.230 (−1.548)	−0.257 (−1.970)	0.075 (0.381)
*R* ^2^	0.446	0.642	0.442	0.618	0.429	0.561	0.521
Adjusted *R*^2^	0.434	0.633	0.43	0.609	0.417	0.55	0.51
*F*	*F*_(5, 235)_ = 37.855, *p* = 0.000	*F*_(6, 234)_ = 70.084, *p* = 0.000	*F*_(5, 235)_ =37.170, *p* = 0.000	*F*_(6, 234)_ = 63.202, *p* = 0.000	*F*_(5, 235)_ = 35.339, *p* = 0.000	*F*_(6, 234)_ = 49.898, *p* = 0.000	*F*_(5, 235)_ = 51.057, *p* = 0.000

** *p < 0.01*,

* *p < 0.05;*

*Figures in parentheses are SEs*.

## Discussion and Conclusions

With the purpose to fill in the identified research gaps, this study was an endeavor to explore the effect of CSR engagement in social media during and after the COVID-19 pandemic with psychological insights. More precisely, with particular attention to the shared brands, this study is concerned with the psychological mechanism of whether and how the behavioral intention of customers could be influenced by their CSR engagement in social media during the damaging coronavirus pandemic.

Through the investigation of the perception of customers on CSR engagement of DiDi (a Chinese representative shared brand) on WeChat, it was found that DiDi engaging in such initiatives during the COVID-19 could bring them positive effects while they are experiencing a market downturn caused by this crisis. This result, therefore, further led evidence to the claims emphasizing the significant role of CSR engagement in a critical period and the great potential of social media for such organizational activities (She and Michelon, 2019; Puriwat and Tripopsakul, 2021). More precisely, with empirical statistics, this study first found that companies with CSR engagement in fact satisfied the self-need of customers, which, in turn, could positively strengthen their identification of customers with themselves even in times of crisis (Bhattacharya and Sen, 2003; Lii and Lee, 2012). Moreover, social media could accelerate the social identification process in that the engagement had been intensified with more accessibility (Fatma et al., 2020). Second, this study also evidenced the partial mediating role of C–C identification between CSR engagement and behavioral intention of customers (Bhattacharya and Sen, 2003; Martínez and Rodríguez del Bosque, 2013). Taking advantage of social media, companies with enhanced C–C identification in fact could optimize brand loyalty, purchase intention, and positive eWOM of their customers. To the best of our knowledge, this study offers an explorative insight into the psychological process concerning the effect of CSR engagement of the shared brands on the behavioral intention of customers during the COVID-19, which is a relatively under-researched topic in the CSR literature.

With regard to the theoretical implication, the findings of this study in fact add to the CSR study from three perspectives. First, this study expanded the discussion of CSR performed by new industries through emerging social platforms (the CSR engagement of DiDi on WeChat). From a review of the study, it indicates that prior studies have typically confined to the CSR practices by traditional industries in traditional messages channels (Brammer et al., 2007; She and Michelon, 2019; Puriwat and Tripopsakul, 2021). How social media interplay with CSR initiatives of emerging industries is relatively an unresearched area (Hu et al., 2019). Second, this study offers further evidence of the necessity of CSR engagement, especially in such a critical period as the COVID-19 pandemic. To comprehensively understand its impact, this study typically investigated CSR practices happening in a critical period. Through empirical statistics, it confirmed the critical role of CSR engagement for business in relation to the behavioral intention of customers while confronted by such a negative external environment as the pandemic COVID-19 (Puriwat and Tripopsakul, 2021). Third, based on the social identity theory, this study dissected the psychological mechanism underlying the effects of CSR engagement on the behavioral intention of customers. Most notably, it is suggested that C–C identification in fact plays a mediating role between CSR engagement and behavioral intention of customers, which corresponds to the claims of Han, H. et al. (2020) and He and Li (2011). Overall, of theoretical concern, this study based on an interdisciplinary perspective in nature is a stepping stone for further studies of CSR engagement occurring in social media.

From a practical standpoint, the findings of this study yield some managerial implications for business practitioners. Regardless of the sudden decrease in the available capitals for business operation during the COVID-19, managers are still suggested to invest in their CSR engagement that in fact can help them win over more economic benefits in the competitive market. In light of the mediating role of C–C identification, it is indicated that companies with CSR engagement are more likely to stimulate their identification, which, in turn, could effectively influence the behavioral intention of customers. During the coronavirus pandemic, there is an observed change happening on the communicative landscape where people are increasingly involved themselves in social media. In this way, managers should utilize social media as a promotional tool that has a great potential to enhance the behavioral intention of customers, thereby increasing their economic returns.

Several limitations of this study also offer directions for future studies. This study has typically focused on the CSR engagement of DiDi happening on WeChat, which restricts the generalizability of the results. Further studies should incorporate other social media platforms and more shared brands for a comprehensive understanding. Second, in response to the real-time external environment, the researcher examined the CSR engagement while the world is experiencing the damaging coronavirus. A longitudinal study is hence suggested to be necessary for making a comparative analysis of the CSR engagement in different periods.

## Data Availability Statement

The original contributions presented in the study are included in the article/supplementary files, further inquiries can be directed to the corresponding author/s.

## Ethics Statement

Ethical review and approval was not required for the study on human participants in accordance with the local legislation and institutional requirements. Written informed consent to participate in this study was provided by the participants' legal guardian/next of kin.

## Author Contributions

YW and WZ together developed the present research idea. YW was responsible for the data collection, data analysis, and the first draft of the manuscript. WZ acted as an instructor throughout the work and contributed to improve the manuscript. All authors contributed to the article and approved the submitted version.

## Conflict of Interest

The authors declare that the research was conducted in the absence of any commercial or financial relationships that could be construed as a potential conflict of interest.

## Publisher's Note

All claims expressed in this article are solely those of the authors and do not necessarily represent those of their affiliated organizations, or those of the publisher, the editors and the reviewers. Any product that may be evaluated in this article, or claim that may be made by its manufacturer, is not guaranteed or endorsed by the publisher.
